# Cost-Effectiveness of Primary Prevention of Stroke in Type 2 Diabetes in the United States: A Microsimulation Analysis

**DOI:** 10.1007/s11606-025-10067-x

**Published:** 2025-12-08

**Authors:** Wen Ye, Xiaqing Jiang, Jing Li, Shihchen Kuo, Christopher J. Becker, William H. Herman, Weizhou Zhang, Lewis B. Morgenstern, Lynda D. Lisabeth

**Affiliations:** 1 Department of Biostatistics, School of Public Health, University of Michigan, Ann Arbor, MI USA; 2 Department of Psychology, University of California San Francisco, San Francisco, CA USA; 3 Department of Internal Medicine, University of Michigan Medical School, Ann Arbor, MI USA; 4 Department of Neurology, University of Michigan Medical School, Ann Arbor, MI USA; 5 Department of Epidemiology, School of Public Health, University of Michigan, Ann Arbor, MI USA

**Keywords:** acute ischemic stroke, cost-effectiveness, prevention, type 2 diabetes

## Abstract

**Background:**

Implementation of guideline-recommended strategies to prevent acute ischemic stroke (AIS) in type 2 diabetes (T2D) remains suboptimal.

**Objectives:**

We evaluated and compared the health impact and cost-effectiveness of improved implementation of guideline-recommended strategies for AIS prevention in patients with T2D in the United States.

**Design:**

We compared scenarios with enhanced implementation of these prevention strategies to the status-quo using a microsimulation model.

**Participants:**

National Health and Nutrition Examination Survey (NHANES) 2015–2018 participants ≥ 45 years of age with T2D and no stroke history.

**Main measures:**

We evaluated stroke-related events, costs, stroke-related quality-adjusted life-years (QALYs), incremental cost-effectiveness ratios, and net health benefit (NHB) from a health system perspective over a 10-year time horizon. A discount rate of 3% per year was applied to costs and QALYs. Costs were expressed in 2022 U.S. dollars.

**Key Results:**

Full implementation of guideline-recommended blood pressure (BP), statin, and aspirin therapies, and smoking cessation would each be cost-saving or highly cost-effective (< $50,000 per QALY-gained). Over 10 years, full implementation of all four of the strategies would prevent 151,000 stroke events, 61,900 deaths from stroke, save $13.4 billion, and produce a nationwide increase of 1,552,000 QALYs (NHB).

**Conclusions:**

Recent attention has focused on the treatment of AIS. We demonstrate that substantial opportunities exist to improve the primary prevention of AIS in Americans with T2D. Providers and payers should prioritize adherence to guidelines for BP, statin and aspirin therapy, and smoking cessation for stroke prevention.

**Supplementary Information:**

The online version contains supplementary material available at 10.1007/s11606-025-10067-x.

## INTRODUCTION

Stroke is a leading cause of morbidity and mortality in people with type 2 diabetes (T2D) and is the single greatest contributor to the cost of T2D^1^. Measuring and improving the quality of care for patients with acute ischemic stroke (AIS) including speed of transfer for thrombolysis and thrombectomy has received much recent attention^2–7^.

Strategies for the primary prevention of AIS have received much less attention. Effective interventions have been recommended by the American Diabetes Association (ADA)^8^ and the American Heart Association/American Stroke Association (AHA/ASA)^9–11^ for the primary prevention of AIS in people with diabetes, but their level of implementation remains suboptimal. Among the 3.7 million U.S. adults with T2D ≥ 45 years of age with no histories of AIS, 30–40% do not meet targets for glycemic or blood pressure (BP) control, 46% are not treated with statins, and ~ 20% continue to smoke^12^. Moreover, the incidence of stroke among people with diabetes remains high. In 2018, 334,000 stroke-related hospital discharges were reported among adults with diabetes^13^. Opportunities exist to improve the primary prevention of AIS in adults with T2D and to reduce morbidity, mortality, and costs^14^.

Over the past decade, the total direct medical costs attributable to diabetes in the U.S. have increased by 35% from $227 billion in 2012 to $307 billion in 2022^15^. Care for people with diabetes in the U.S. accounts for one in four health care dollars^15^. Given the increasing cost of T2D and persistent disparities in care, priorities for resource allocation should be established. Curative health services are expensive and their limited availability may exacerbate disparities. In contrast, preventive health care is generally inexpensive and available and could potentially benefit a far larger population^16^.

Although studies have demonstrated that many of the strategies recommended by ADA and AHA/ASA to prevent AIS among individuals with T2D are cost-effective^17–20^, the evaluations have focused on single interventions. Policy-making requires evidence about the cost-effectiveness of numerous policy-relevant interventions^21,22^. No study has compared all of the guideline-recommended preventive strategies for AIS in a nationally representative population. Doing so will allow policy-makers to determine which measures have the highest value and should be prioritized for stroke prevention in the U.S. population with T2D.

The primary aim of our study is to use a computer microsimulation model to estimate the clinical outcomes, health care costs, and cost-effectiveness of enhanced implementation of seven guideline-recommended primary stroke prevention strategies compared to the current level of care among U.S. adults with T2D (Table 1). The results are intended to inform health policy and resource allocation for the primary prevention of AIS in the United States.

**Table 1:** Recommendations for preventing stroke in type 2 diabetes patients without stroke history

	Recommendation	Proportion (95% CI) of national population achieving recommended strategies baseline NHANES 2015–2018 (Weighted)
Well-controlled HbA1c^1,29,31^	Target of < 7% (for patients aged 45–64 years) or < 7.5% (for patients aged ≥ 65 years)	57.6% (53.2%, 60.1%)
Well-controlled BP^9,31^	Target of < 140/90 mm Hg and treatment with an ACE inhibitor or angiotensin receptor blocker as the first-line medication	70.7% (66.8%, 74.8%)
Statin treatment^31^	For primary prevention, moderate-dose statin therapy is recommended for those 40 years and older. In patients with diabetes who are at higher risk, especially those with multiple ASCVD risk factors, or with ASCVD risk > 20%, or aged 50–70 years, it is reasonable to prescribe high-intensity statin therapy. Moderate-intensity statin therapy is recommended in patients with diabetes who are 75 years or older	60.0% (55.6%, 64.4%)
Treatment with Aspirin/clopidogrel^31^	Recommendations for using aspirin as primary prevention include both men and women aged ≥ 50 years with diabetes and at least one additional major risk factor (family history of premature ASCVD, hypertension, dyslipidemia, smoking, or chronic kidney disease/albuminuria) who are not at increased risk of bleeding	7.9% (3.3%, 12.5%)
Non-smoking^31^	A diabetes patient should quit smoking	81.1% (77.5%, 84.7%)
Well-controlled BMI^9^	Among overweight (BMI = 25–29 kg/m^2^) and obese (BMI > 30 kg/m^2^) individuals, weight reduction is recommended for reducing the risk of stroke	10.8% (8.2%, 13.4%)
Warfarin/NOAC treatment^9^	Treatment with warfarin for patients with atrial fibrillation (AFib) at high risk for stroke (defined as a CHA_2_DS_2_-VASc score > = 2)	52.4% (29.6%, 75.3%)

## METHODS

### The Michigan Model for Diabetes

**Figure 1:**
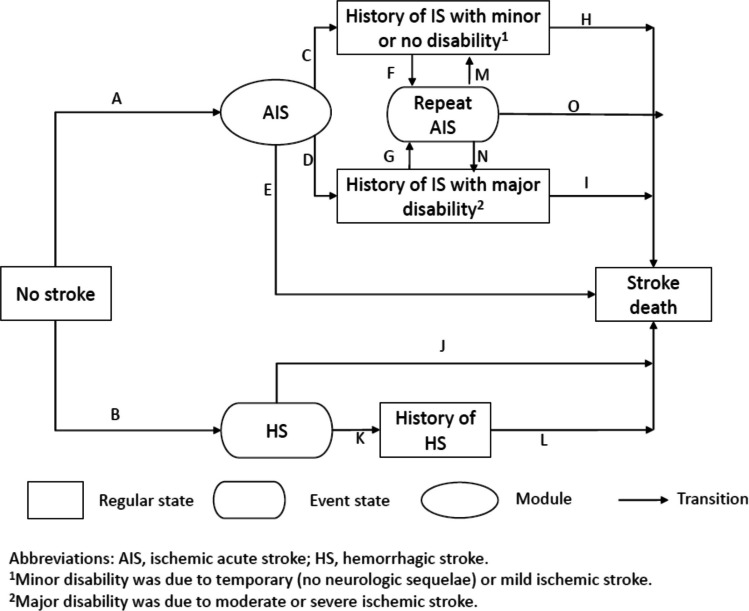
Schematic representation of model structure of the MMD 3.2 stroke sub-model.

The Michigan Model for Diabetes (MMD)^23–26^ is a validated microsimulation model for T2D. Disease progression is based on six discrete-time discrete-event sub-models that simulate diabetes-related complications, major comorbidities, and death due to diabetes-related and non-diabetes-related causes. Transition probabilities are functions of the characteristics of individuals, risk factor levels, and current disease and treatment states. The model estimates the direct medical costs and the health-related quality-of-life (HRQOL) for each simulated individual. Figure 1 shows the structure of the stroke sub-model.

All simulations were performed using the MMD version 3.2, which was programmed in R and is available to the public^27^. The validation results of MMD 3.2 can be found in Appendix A.

MMD 3.2 explicitly models diabetes management strategies and stroke prevention strategies recommended by ADA and AHA/ASA through a treatment module. At the beginning of each simulation year, the treatment module recommends treatment adjustments (or no changes) based on each individual’s risk factor levels (Hemoglobin A1c [HbA1c], BP, lipids), medical history (myocardial infarction [MI], stroke, atrial fibrillation), and other criteria (e.g. stroke clinical risk score^28^). Each simulated individual adopts or does not adopt treatment recommendations based on pre-assigned adherence parameters. For those who adopt treatment recommendations, their risk factors change based on treatment effects reported in the literature. MMD 3.2 also models changes in risk factors related to aging and the natural history of each condition. By adjusting adherence at the population level, we are able to adjust the proportion of individual in the population achieving recommended risk factor control and stroke prevention strategies, and quantify the new treatment costs related to the recommendations implemented.

The treatment module explicitly models strategies to achieve optimal glycemic control (from intensive lifestyle intervention to intensive insulin therapy), BP control (from one half dose of a single medication to full dose of 3 blood pressure medications), and lipid management (from moderate dose statin therapy to high dose statin therapy). This allows us to assess the impact of each strategy relative to the baseline risk factor and treatment levels in the population. Details about MMD 3.2 can be found in its User Manual online^27^.

### Stroke-related Costs and Health Outcomes

We simulated health care costs associated with treatments, stroke, and death over a 10-year simulation time horizon. Estimated costs included the costs of medications, procedures, devices, physical therapy, occupational therapy, and death. All costs were expressed in 2022 U.S. dollars. We also assessed health outcomes including AIS events, stroke-attributed death, and stroke-related quality-adjusted life-expectancy (QALE) expressed as quality-adjusted life-years (QALYs) over a 10-year simulation time horizon. We calculated incremental cost-effectiveness ratios (ICERs) as the additional cost required to achieve one unit of stroke-related QALE for each enhanced prevention scenario vs. the status-quo scenario. We considered strategies with an ICER < $100,000 per QALY-gained to be cost-effective^34,35^. We calculated the incremental net health benefit (NHB) in QALYs, as stroke-related QALYs gained minus incremental cost/cost effectiveness threshold, which accounts for both the QALYs gained from the health effects of an intervention and QALYs lost as the opportunity cost of additional spending.

### Base-Case Analyses

We used NHANES data from 2015 through 2018 to identify individuals with T2D 45 years of age and older without histories of stroke. Using survey-weighted analyses, we estimated the proportion of those individuals achieving each of the seven guideline-recommended strategies for stroke prevention (Table 1). These included HbA1c control, BP control, statin treatment, aspirin/clopidogrel treatment, non-smoking, achievement of recommended body mass index (BMI), and anticoagulant treatment (warfarin/non-vitamin K antagonist oral anticoagulants [NOACs]) for patients with atrial fibrillation at high risk for stroke. Because NHANES did not describe the atrial fibrillation status of individual participants, we imputed atrial fibrillation status based on information from NHANES and from the literature.

We used the NHANES population’s individual-level characteristics including demographics, medication use, atherosclerotic cardiovascular disease risk-factor levels, and diabetic complications and comorbidities, to populate the MMD 3.2. Because medications, management strategies, and technologies for T2D are changing rapidly and are likely to be outdated in 10 years, we chose to use a 10-year time horizon for our base-case scenario. Simulation was used to project outcomes for the U.S. population ≥ 45 years of age with T2D and no history of stroke for the period from 2018 through 2028. In the status-quo scenario, we adjusted the population level adherence to six of the seven recommended preventive strategies (well-controlled HbA1c, well-controlled BP, statin treatment, treatment with aspirin/clopidogrel, non-smoking, and warfarin/NOAC treatment) to make the simulated percentage achieving each recommendation the same as the average national rate between 2015 and 2018 as shown in Table 1. The exception was for BMI control. We kept BMI changes over time as they were programmed in the MMD, which reflect the changes associated with aging and anti-hyperglycemic treatment. In MMD 3.2, individuals who were designated as being adherent to a specific preventive strategy adopted that strategy when they met the recommended threshold for that treatment.

We then simulated enhanced treatment scenarios. In the first seven scenarios, we optimized adherence to each of the recommendations one at a time (Table 2). In the eighth prevention scenario, we implemented all the strategies that were found to be cost-saving or highly cost-effective (incremental cost-effectiveness ratio [ICER] < $50,000 per QALY-gained) individually. In the next prevention scenario, we implemented all the strategies that were found to be cost-saving or cost-effective (incremental cost-effectiveness ratio [ICER] < $100,000 per QALY-gained) individually. In the final scenario, we implemented all seven of the prevention strategies regardless of the individual cost-effectiveness.

**Table 2:** Enhanced prevention scenarios in main analyses

Enhanced HbA1c control^1,8,29,30^	100% adherence to antihyperglycemic treatment enhancement to achieve target HbA1c level. At the beginning for each simulation time interval (year), all patients whose HbA1c is above the recommended target are not on intensive insulin treatment enhance their antihyperglycemic treatment to the next level. Note that this is not equivalent to achieving the recommended threshold at all-time points
Enhanced blood BP control^9,31^	100% adherence to BP medication enhancement to achieve target BP level. At the beginning for each simulation time interval, all patients whose SBP/DBP is above the recommended target and is taking fewer than 3 full dose antihypertension medications enhance their antihypertensive treatment to the next level
Enhanced Statin treatment^31^	100% of the population take a statin at the recommended level
Enhanced treatment with aspirin/clopidogrel^31^	100% of patients who are aged 50–70 years with diabetes and at least one additional major risk factor take aspirin
Enhanced smoking cessation^32,33^	Smoking Cessation: All smokers attend behavioral interventions for smoking cessation and 12% successfully quit smoking at the beginning of the simulation period
Enhanced BMI control^9^	All patients with BMI > 25 kg/m^2^ participate in weight loss program to lose 5% of their body weight at the beginning of the simulation
Enhanced anti-coagulant treatment with NOAC^9^	100% of patients with AFib at high risk for stroke (defined as a CHA_2_DS_2_-VASc score > = 2) who were not on warfarin or NOAC at baseline will be on NOAC
Enhanced multiple preventions 1	A combination of prevention strategies that are shown to be highly cost-effective or cost-saving when tested alone
Enhanced multiple preventions 2	A combination of prevention strategies that are shown to be cost-effective or cost-saving when tested alone
All preventions enhanced	A combination of all prevention strategies

One hundred multiple imputation datasets were used to deal with the missing observations in the NHANES population at baseline and to account for uncertainty due to missing data. We ran each of the 100 imputed baseline populations 1,000 times to decrease Monte Carlo uncertainty in the means of the outcomes. When summarizing the simulation results, survey sampling weights were used to derive results that can be generalized to the U.S. population with T2D. Important costs and utility penalties used in the base-case analysis are show in Appendix B.

### Sensitivity Analyses

We assessed efficacy with one-way sensitivity analyses that used lower and upper uncertainty boundaries for parameters related to efficacy (Appendix C). These parameters included the hazard ratio related to a 1% decrease in HbA1c, a 10 mmHg decrease in systolic blood pressure (SBP), a 1 unit decrease of lipid ratio (total cholesterol/high-density lipoprotein), and relative risk reductions related to aspirin and NOAC treatment. In early 2023, the three major insulin manufacturers—Eli Lilly, Novo Nordisk, and Sanofi—lowered the list prices for some of their insulin products^36^. We conducted a sensitivity analysis for glycemic management using the lower costs of insulin treatment. Finally, recognizing that full implementation (100% adherence to all recommended strategies) of the seven prevention strategies is unlikely, we conducted sensitivity analyses assuming 50% and 25% improvement in adherence to each of the strategies. We also carried out a lifetime simulation (50-year time horizon).

The impact inventory^37^ for this cost-effectiveness analysis is provided in Appendix D. This model-based cost-effectiveness analysis was conducted in compliance with the Consolidated Health Economic Evaluation Reporting Standards (Appendix E)^38^. All analyses involved secondary analyses of publicly available, de-identified data. All analyses were performed from a health system/payer perspective. Future costs and QALYs were discounted at 3% per year.

## RESULTS

Baseline characteristics of the U.S. population ≥ 45 years of age with T2D and no history of stroke are shown in Appendix F. A total of 1,232 NHANES participants were included. Mean age was 65.1 year (SD, 9.8) and 55.8% were men.

### Base-Case Analyses

Table 3 shows the results for the base-case analysis. Among the seven preventive strategies, BP control, statin treatment, and aspirin treatment were cost-saving, smoking cessation, and weight loss were highly cost-effective or cost-effective. Mean trajectories of HbA1c, SBP, and BMI, and percent adherence to recommended antihyperglycemic treatment, antihypertensive treatment, and weight control recommendations with the three corresponding enhanced scenarios are compared to the status-quo scenario in Appendix G.

**Table 3:** Base-Case estimate of population-level incremental health outcomes and nhb and cost-effectiveness of primary prevention strategies for patients with type 2 diabetes and no history of stroke at age 45 years and older (2018–2028).* (Optimal vs. Status Quo)

Strategy	Stroke events averted No. (95% UI) |||| (in 1000)	Stroke attributed death averted No. (95% UI) |||| (in 1000)	Incremental total stroke cost (95% UI) |||| *Billions of $*		Incremental stroke-related QALYs (95% UI) |||| (in 1000)	Incremental NHB (in QALYs) (95% UI) |||| (in 1000)	ICER
Well controlled HbA1c^§^	28.2	12.0	251.3	- 38		−2,552	NA
	(26.3 to 30.1)	(10.7 to 13.2)	(250.9 to 251.8.2)		(−47 to −30)	(−2,579 to −2,545)	
Well controlled BP^‡^	73.1	31.2	−13.9	327		467	Cost Saving
	(71.2 to 74.9)	(29.9 to 32.4)	(−14.4 to −13.4)		(319 to 335)	(457 to 476)	
Statin treatment^||^	24.5	7.8	−0.97		1,254	1,264	Cost Saving
	(22.6 to 26.4)	(6.6 to 9.1)	(−1,672 to −276)		(1,237 to 1,271)	(1,245 to 1,282)	
Aspirin treatment^¶^	56.6	23.8	−5.5		86	141	Cost Saving
	(54.7 to 58.5)	(22.6 to 25.1)	(−5.9 to −4.9)		(78 to 92)	(131 to 150)	
Smoking cessation^**^	9.1	3.4	0.8		37	29	21,712
	(7.2 to 11.0)	(2.2 to 4.7)	(0.3 to 1.3)		(28 to 45)	(19 to 38)	
Weight Loss^†^	59.8	22.4	54.1		555	14	97,393
	(57.9 to 61.7)	(21.2 to 23.7)	(53.6 to 54.6)		(547 to 563)	(4.9 to 24)	
NOAC treatment‡‡	84.8	32.0	13.9		99	−39	139,453
	(82.9 to 86.7)	(30.8 to 33.3)	(13.4 to 14.4)		(91 to 108)	(−49 to −30)	
Multiple preventions 1^§§^	151	61.9	−13.4	1,418		1,552	Cost Saving
	(149 to 153)	(60.7 to 63.2)	(−13.9 to −12.9)		(1,410 to 1,426)	(1,543 to 1,562)	
Multiple preventions 2^ s^	202	81.1	43.0		1,940	1,511	22,156
	(200 to 203)	(79.8 to 82.3)	(42.5 to 43.5)		(1,933 to 1,948)	(1,501 to 1,520)	
All preventions enhanced	300	118.2	316.0		1,914	−1,246	165,086
	(298 to 302)	(117.0 to 119.4.5)	(315.6 to 316.5)		(1,906 to 1,922)	(−1,255 to −1,237)	

* Data were calculated with the use of the Michigan Model for Diabetes on the basis of a simulation of the Recommendation of ADA (2022), AHA/ASA (2014) for preventing stroke in type 2 diabetes patients. A status quo simulation provided a projection of stroke events, costs, and quality-adjusted life-years (QALYs) for the U.S. adult population between the ages of 45 years and older during the period from 2018 through 2028, on the assumptions that 1) the adherence level to each of the stroke prevention recommendation remain at the level observed in 2015–2018 (Table 1). ICER denotes incremental cost-effectiveness ratio. † Weight Loss: 5% reduction for all with BMI > 25 at the beginning of simulation period. ‡ Well Controlled blood pressure (BP): 100% compliance to medication enhancement for blood pressure with enhancement threshold =140mmhg for SBP. § Well Controlled A1c: 100% compliance to medication enhancement with enhancement threshold = 7% for patients aged 45–64 years and = 7.5% for patients aged >=65 years. ¶ Aspirin treatment: All patients whose 10-year ASCVD risk >10% start taking aspirin in the year when this criteria is met. || Statin treatment: All patients 100% start taking statin at the beginning of simulation period. ** Smoking Cessation: All smokers attend behavior intervention for smoking cessation and 12% successfully quit smoking at the beginning of simulation period. †† Warfarin treatment: All patients with atrial fibrillation (AFib) at high risk for stroke (defined as a CHA_2_DS_2_-VASc score >=2) start taking warfarin in the year when this criteria is met. ‡‡ NOAC treatment: All patients with atrial fibrillation (AFib) at high risk for stroke (defined as a CHA_2_DS_2_-VASc score >=2) start taking NOAC in the year when this criteria is met. §§ Implementing two cost-saving strategies (well controlled BP, aspirin treatment, statin treatment) and one highly cost-effective strategy (smoking cessation). §§§ Implementing two cost-saving strategies (well controlled BP, aspirin treatment, statin treatment) and two cost-effective strategies (smoking cessation, and weight loss). |||| 95% UI are from 100,000 simulations

Optimal BP control would prevent 73,100 stroke events, save $13.9 billion, and result in an increase of 467,000 QALYs (NHB) nationwide over ten years (Table 3). Aspirin treatment would prevent 56,600 strokes, save $5.5 billion and result in an additional 141,000 QALYs (NHB) over ten years (Table 3). Full implementation of statin treatment would prevent 24,500 stroke events, save $0.97 billion, and result in an incremental NHB of 1,264,000 QALYs (Table 3).

Having all smokers attend behavioral interventions for smoking cessation would be highly cost-effective with an ICER of $21,712 per stroke-related QALY-gained over ten years. In addition, it would increase NHB by 29,000 QALYs (Table 3). Reducing BMI would be cost-effective with an ICER at $97,393 per stroke-related QALY-gained over ten years, with potential improvement in NHB of 14,000 QALYs (Table 3).

Full implementation of the two cost-saving prevention strategies (BP control and aspirin treatment) and the two highly cost-effective prevention strategies (statin treatment and smoking cessation) together would prevent 151,000 stroke events, 61,900 deaths from stroke, save $13.4 billion, and produce a nationwide increase of 1,552,000 QALYs (NHB) over ten years (Table 3). Full implementation of the five cost-saving or cost-effective prevention strategies would be cost-effective with an ICER at $22,156 per stroke-related QALY-gained and improve NHB by 1,511,000 QALYs (Table 3).

Improving HbA1c control could prevent 28,200 strokes but decrease stroke-related quality adjusted life expectancy (QALE) by 38,000 QALYs, and cost $251.3 billion over ten years (Table 3). The ICER for enhanced anti-coagulant treatment with NOACs would be $139,453 per stroke-related QALY-gained, higher than the cost-effectiveness threshold of $100,000 per QALY-gained. Full implementation of NOAC treatment would lead to a decrease in NHB by 39,000 QALYs over ten years (Table 3). Full implementation of all seven strategies would not be cost-effective.

### Sensitivity Analyses

Using one-way sensitivity analyses, we assessed the impact of our assumptions about the efficacy and cost of the prevention strategies. No one-way sensitivity analysis results would change our conclusions based on the base-case analyses (Appendix C). When we used the lower costs for insulin treatment^36^, the cost per person for improved HbA1c control decreased to $6,618 per person over ten years (Table 2 in Appendix C). However, it would still cost $124 billion nationwide. When we assumed 50% improvement from the status-quo, the conclusion remained consistent with the base-case analysis (Table 3 in Appendix C). When we assumed 25% improvement from the status-quo, BP control and aspirin treatment would be cost-saving, statin treatment and smoking cessation would be highly cost-effective, but the ICER for weight control would be slightly higher than the cost-effectiveness threshold (Table C4 in Appendix C). When enhancing BP control, aspirin treatment, statin treatment, and smoking cessation at the same time, 25% improvement and 50% improvement would result in an incremental NHB of 491,000 and 927,000 QALYs, respectively (Table C3 and C4 in Appendix C).

Over a lifetime time horizon (Table C5 in Appendix C), BP control and aspirin treatment would still be cost-saving, statin treatment, smoking cessation, and NOAC treatment would be highly cost-effective, and weight control would be cost-effective.

## DISCUSSION

Full nationwide implementation of guideline-recommended strategies for BP control, statin therapy, aspirin therapy, and smoking cessation, and combining these four strategies could avert a large number of AIS events and stroke-attributed deaths, and be highly cost-effective or even cost-saving. An advantage of our approach is that we have taken current levels of treatment into account and avoided making arbitrary and potentially unrealistic assumptions about how much one can improve risk factor levels.

Zhu et al. (2024)^7^ conducted a cost-effectiveness analysis to prioritize ten AHA/ASH-endorsed quality measures for AIS management. They showed that for an annual AIS incidence cohort in the U.S. (approximately 530,700 individuals), the gain in life expectancy associated with full implementation of all ten quality measures would range from 117 QALYs for time to intravenous tissue-type plasminogen activator [tPA] to 10,814 QALYs for early carotid imaging in terms of incremental NHB over a lifetime for these AIS patients.

Assuming that the impact of these quality measures are independent as assumed by Zhu et al. (2024)^7^and considering that approximately 22% of AIS patients have diabetes^39^, full implementation of these 10 measures would accrue 54,140 QALYs (NHB) for a 10-year incidence cohort of AIS patients with T2D over their lifetimes. This is only 3% of the estimated maximum gain associated with full implementation of the guideline-recommended stroke prevention strategies (BP control, statin therapy, aspirin therapy, and smoking cessation) that we reported (1,552,000 QALYs (NHB) over 10 years).

Over ten years, enhanced statin therapy would be cost saving and result in the greatest improvement in QALE compared to the status-quo. This improvement likely reflects the benefits of statin therapy on coronary heart disease morbidity and mortality (the number of strokes averted was 16,700 and the corresponding number of MI events averted was 363,000). Over a lifetime time horizon, full enhancement of statin treatment would not be cost saving, but highly cost-effective (Table C5 in Appendix C). It is interesting that under the enhanced scenarios, there is a larger number of stroke events than under the status-quo scenario. This is likely due to statin’s large benefit on coronary heart disease morbidity and mortality, which results in longer life expectancy and opportunity to experience stroke events. Statin use in this population increased from 30% in 2001 to almost 60% in 2018^12^. Further increases in statin use should be encouraged as an effective strategy to improve QALE.

Of the seven prevention strategies, NOAC treatment for atrial fibrillation averted the most strokes (83,100) over ten years. Interestingly, the improvement in QALE over 10 years was not large (0.0048 per person). Although enhancing NOAC treatment for atrial fibrillation could substantially decrease the number of stroke events, it was not cost-effective. This is probably due to the fact that our analysis adopted a 10-year time horizon, atrial fibrillation prevalence increases with age, and many untreated people with atrial fibrillation are 70 years of age and older. Competing death thus limits the benefit of NOAC therapy in terms of QALE. A sensitivity analysis that took a lifetime time horizon showed that enhancing adherence to NOAC treatment would be highly cost-effective over a lifetime (ICER: $29,828 per QALY gained) (Table C5 in Appendix C).

In 2015–2018, 7.9% of patients with diabetes aged ≥ 50 years with at least one additional major cardiovascular risk factor were taking aspirin (Table 1). Enhancing aspirin treatment for this population would improve QALE and save $5.5 billion in direct medical costs over ten years. Due to risk of gastrointestinal bleeding, not all patients should take aspirin, but there remains an opportunity to increase appropriate aspirin use. The results of a sensitivity analysis showed that partial implementation of aspirin use also produce substantial gains in QALE for the U.S. population with T2D (Appendix C).

We found that improving glycemic control would lead to a large increase in healthcare expenditures and only a slight decrease in QALE due to the high cost of insulin, the disutility associated with injections and monitoring, and the increased incidence of hypoglycemia. This finding persisted when lower prices of insulin were applied. Although both acute and chronic hyperglycemia have been associated with increased incidence and morbidity from stroke, intensive lowering of glucose levels has generally not been shown to prevent macrovascular events including stroke^40,41^.

### Limitations

Like all computer-simulation model-based analyses, ours relied on multiple assumptions and data derived from multiple sources. The MMD 3.2 uses a yearly simulation interval. Risk factors and treatments are updated each year. This does not allow for multiple annual adjustments to quickly achieve optimal glucose and blood pressure control. Because of this, our results are probably conservative in demonstrating the benefits of BP and glucose control. In addition, we only tested conventional treatment options and did not assess the impact of newer therapies. For example, we used lifestyle intervention for weight control and did not evaluate pharmacotherapy or metabolic surgery. For glycemic control, we did not evaluate the impact of the newer antihyperglycemic medications. A recent meta-analysis^41^ found that treatment with glucagon-like peptide-1 receptor agonists (GLP1-RAs) and thiazolidinediones reduce the risk of stroke, while other glucose-lowering medications lack evidence for an effect on stroke risk. Further work is warranted to study cost-effectiveness of stroke prevention with GLP1-RAs, thiazolidinediones, and weight management with metabolic surgery.

We did not consider the cost of system-level interventions (e.g., changes in clinical practice, improvements in patients’ access to care, addressing social determinants of health) to promote these guideline-recommended strategies. However, based on our results, enhanced implementation of four cost-saving or highly cost-effective strategies would save approximately $13 billion over 10 years. Spending less than this amount on system-level intervention to promote these four strategies over 10 years would still be cost saving.

Siloed health-care delivery systems, underutilization of team-based care, and overreliance on individual clinicians to remember the myriad of guideline-directed therapies that a given patient should receive are barriers to improving diabetes management in the U.S.^42^. Egan et al. (2021)^43^ showed that hypertension control in the United States to a target of < 140/< 90 mm Hg was significantly lower between 2015 and 2018 than between 2009 and 2014 among all adults with the condition, likely related to the increase in hypertension awareness, treatment, and treatment effectiveness observed in the same time period. Quality improvement programs including MAP BP^44^ and those implemented by Kaiser Permanente^45^can facilitate rapid and sustained improvements in hypertension control. A systemic review of quality improvement programs for adults living with diabetes showed that clinics can improve their diabetes care by engaging case management, team changes, patient education and patient self-management^46^.

### Generalizability

We evaluated seven prevention strategies in a nationally representative U.S. population. To answer similar questions in other countries or areas in the world, one needs to consider the specific level of achievement of T2D management goals and the costs of treatment in country and area. The levels of risk factor control vary in different parts of the world^42^. For example, based on the DISCOVER study^46^ the percentage of T2D patients with SBP < 140 mmHg ranged from 56.5% in Europe to 76.1% in South-East Asia. The cost-effectiveness and priorities of the stroke prevention strategies would also vary in different regions.

## CONCLUSIONS

Enhanced implementation of guideline-recommended primary prevention strategies for stroke including BP control, statin treatment, aspirin treatment, and smoking cessation for the U.S. population ≥ 45 years of age with T2D and no stroke history could potentially avert a large number of stroke events and stroke-attributed deaths, improve stroke-related QALE, and reduce health care costs. Better implementation of strategies for the primary prevention of AIS may be more impactful than the treatment of AIS. Quality improvement initiatives for AIS prevention in the U.S. population with T2D are needed.

## Supplementary Information

Below is the link to the electronic supplementary material.

ESM1 (DOCX 205 KB)

## Data Availability

Details about the Michigan Model for Diabetes (MMD) and a RShiny App of MMD are available at https://michigandiabetesmodelinggroup.github.io/Software_App/MMD_3_2. National Health and Nutrition Examination Survey NHANES data is available through public download on the CDC website, which includes data files and documentation for free access.
