# Supplementary material for: Cost-Effectiveness of Primary Prevention of Stroke in Type 2 Diabetes in the United States: A Microsimulation Analysis
Source: J Gen Intern Med. 2025 Dec 8;41(7):1928–36. doi: 10.1007/s11606-025-10067-x (PMC13176362; doi:10.1007/s11606-025-10067-x)
Supplement: Supplementary file 1 — (DOCX 205 KB) [file 11606_2025_10067_MOESM1_ESM.docx]

**SUPPLEMENTARY MATERIALS**

Appendix A. Model Validation

Appendix B. Important Costs and Utility Penalties Used in the Base-case Analysis

Appendix C. Sensitivity Analyses

Appendix D. Impact inventory for components considered in the cost-effectiveness analyses

# Appendix E. CHEERS 2022 Checklist

Appendix F. Baseline Characteristics of the Simulation Population

Appendix G. Additional Base-case Analysis Results

**Appendix A. Model Validation**

We validated our model against the placebo arm in the EMPA-REG OUTCOME trial^1^.

To simulate outcomes for the placebo group during the study period, adherence to treatment was adjusted to allow risk factor and biomarker levels to match those reported from the study over time (EMPA-REG OUTCOME trial). We ran the MMD simulations for the mean length of follow-up reported by the trial (three years). For each treatment group, we simulated the number of participants randomized in the trial (N = 2333 for the placebo group and N = 4687 for the pooled empagliflozin group). We repeated the simulations 100 times.

| Table A1. Model validation results | | |
| --- | --- | --- |
|  | Study outcome (number of events per 1000 person year) | Simulation Outcome (number of events per 1000 person year) |
| MI | 19.3 | 19.5 |
| Stroke | 10.5 | 10.4 |
| CVD Death | 20.3 | 22.3 |
| Total Death | 28.6 | 30.0 |
| Primary outcome^*^ | 43.9 | 41.1 |

*Primary outcome: death from cardiovascular causes, nonfatal myocardial infarction, or nonfatal stroke

^1^ Zinman B, Wanner C, Lachin JM, et al. EMPA-REG OUTCOME Investigators. Empagliflozin, cardiovascular outcomes, and mortality in type 2 diabetes. N Engl J Med 2015;373:2117–28.

**Appendix B. Important Costs and Utility Penalties Used in the Base-case Analysis**

| Supplementary Table B1. Important costs and utility penalties used for the base-case analysis | | | | | |
| --- | --- | --- | --- | --- | --- |
|  | Base value | |  | | |
| Cost (2014 US dollar) |  |  | |  |  |
| **Event-year cost of ischemic stroke^*^** |  | |  | | |
| Standard medical therapy without rtPA or EVT | $55,278 per event | | O’Brien et al. 2003, Patil et al. 2009 | | |
| **Ongoing cost of ischemic stroke after ischemic stroke^*^** |  | |  | | |
| With no/minor disability after ischemic stroke | $10,311 per year | | O’Brien et al. 2003, O’Brien and Gage 2005, Freeman et al., 2011, Harrington et al. 2013, Nguyen et al. 2016 | | |
| With major disability after ischemic stroke | $26,585 per year | |  |  |  |
| **Hemorrhagic stroke** |  | |  | | |
| Event-year cost of hemorrhagic stroke | $153,633 per event | | O’Brien et al. 2003, O’Brien and Gage 2005, Freeman et al., 2011, Harrington et al. 2013, Nguyen et al. 2016 | | |
| Ongoing cost of hemorrhagic stroke after hemorrhagic stroke | $34,268 per year | |  |  |  |
|  |  | |  | | |
| **Medication cost** |  | |  | | |
| Warfarin | $471.8 per year | | Including drug and monitoring costs | | |
| NOAC | $5,495 per year | | RED BOOK Online-Micromedex Solutions^§^ | | |
|  |  | |  | | |
| Aspirin | $4 per year | | RED BOOK Online-Micromedex Solutions^§^ | | |
| Hypertensive treatment |  | |  | | |
| Level 1: One med with half dose | $22 per year | | RED BOOK Online-Micromedex Solutions^§^ Details in MMD manual | | |
| Level 2: One med with full dose | $29 per year | |  |  |  |
| Level 3: Two meds, including 1^st^ med with full dose and 2^nd^ med with half dose | $62 per year | |  |  |  |
| Level 4: Two meds, including 1^st^ med with full dose and 2^nd^ med with full dose | $44 per year | |  |  |  |
| Level 5: Three meds, including 1^st^ med and 2^nd^ med with full dose, and 3^rd^ med with half dose | $77 per year | |  |  |  |
| Level 6: Three meds, including 1^st^ med, 2^nd^ med, and 3^rd^ med with full dose | $84 per year | |  |  |  |
| Treatment for hyperglycemia |  | |  | | |
| Level 1: Intensive lifestyle (IL) | $1,482 per year | | RED BOOK Online-Micromedex Solutions^§^ Details in MMD manual | | |
| Level 2: One non-insulin medication | $502 per year | |  |  |  |
| Level 3: Two non-insulin medications | $936 per year | |  |  |  |
| Level 4: Add basal insulin^1^ | $5,504 per year | |  |  |  |
| Level 5: Intensify insulin therapy (basal/bolus)^2^ | $16,488 per year | |  |  |  |
| Statin |  | |  | | |
| Moderate potency | $135 per year | | RED BOOK Online-Micromedex Solutions^§^ Details in MMD manual | | |
| High potency | $135 per year | |  |  |  |
|  |  | |  | | |
| Weight watcher program cost | $386 per year | | Finkelstein and Kruger 2014 | | |
| Smoking Cessation program cost | $398 (one time cost) | | Rumberger et al., 2010 | | |
|  |  | |  | | |
| Stroke related utility penalty |  |  | |  |  |
| Ischemic stroke (IS) with no/minor disability after IS | -0.01 | | Joundi et al. 2022, Rebchuk et al. 2020, Butsing et al., 2025 | | |
| Ischemic stroke (IS) with major disability after IS | -0.17 | |  |  |  |
| History of hemorrhagic stroke (HS) | -0.20 | |  |  |  |
|  |  | |  | | |
| Parameters related to treatment effect |  |  | |  |  |
|  |  | |  | | |
| Log(hazard ratio) related to 1 unit of lipid ratio (Total cholesterol/HDL) | 0.113 | | Clarke et al., 2004 | | |
| Log(hazard ratio) related to 1% of increase in A1c | 0.128 | |  |  |  |
| Log(hazard ratio) related to 10 unit of increase in SBP | 0.276 | |  |  |  |
| Relative risk for effect of Warfarin | 0.32 | | Kahwati et al., 2022 | | |
| Relative risk for effect of Warfarin | 0.256 | | Kahwati et al., 2022 | | |
| Relative risk for effect of Aspirin | 0.88 | | ASCEND Study Collaborative Group, 2018 | | |
| ^#^The utility decrement of ischemic stroke with major disability after ischemic stroke was based on the utility decrement of stroke with residual which was stroke with any persistent difficulty speaking, any weakness, or difficulties performing daily activities or working.  ^§^ Micromedex 2.0 (electronic version). Truven Health Analytics, Greenwood Village, Colorado, USA. Available at: <https://www.micromedexsolutions.com/mircomedex2/librarian> (accessed: 2/17/2023) | | | | | |

ASCEND Study Collaborative Group; Bowman L, Mafham M, Wallendszus K, Stevens W, Buck G, Barton J, Murphy K, Aung T, Haynes R, Cox J, Murawska A, Young A, Lay M, Chen F, Sammons E, Waters E, Adler A, Bodansky J, Farmer A, McPherson R, Neil A, Simpson D, Peto R, Baigent C, Collins R, Parish S, Armitage J. Effects of Aspirin for Primary Prevention in Persons with Diabetes Mellitus. N Engl J Med. 2018 Oct 18;379(16):1529-1539. doi: 10.1056/NEJMoa1804988. Epub 2018 Aug 26. PMID: 30146931.

Butsing N, Voss JG, Keandoungchun J, Thongniran N, Griffin MTQ. Changes of health-related quality of life within 6 months after stroke by clinical and sociodemographic factors. Sci Rep. 2025 Jan 2;15(1):416. doi: 10.1038/s41598-024-84454-5. PMID: 39747957; PMCID: PMC11695920.

Clarke PM, Gray AM, Briggs A, Farmer AJ, Fenn P, Stevens RJ, Matthews DR, Stratton IM, Holman RR; UK Prospective Diabetes Study (UKDPS) Group. A model to estimate the lifetime health outcomes of patients with type 2 diabetes: the United Kingdom Prospective Diabetes Study (UKPDS) Outcomes Model (UKPDS no. 68). Diabetologia. 2004 Oct;47(10):1747-59. doi: 10.1007/s00125-004-1527-z. Epub 2004 Oct 27. PMID: 15517152.

Coffey JT, Brandle M, Zhou H, Marriott D, Burke R, Tabaei BP, Engelgau MM, Kaplan RM, Herman WH. Valuing health-related quality of life in diabetes. Diabetes Care 2002;25:2238-2243.

Finkelstein EA, Kruger E. Meta- and cost-effectiveness analysis of commercial weight loss strategies. Obesity (Silver Spring). 2014 Sep;22(9):1942-51. doi: 10.1002/oby.20824. Epub 2014 Jun 24. PMID: 24962106.

Freeman JV, Zhu RP, Owens DK, et al. Cost-effectiveness of dabigatran compared with warfarin for stroke prevention in atrial fibrillation. Ann Intern Med 2011;154:1-11.

Harrington AR, Armstrong EP, Nolan PE Jr, Malone DC. Cost-effectiveness of apixaban, dabigatran, rivaroxaban, and warfarin for stroke prevention in atrial fibrillation. Stroke 2013;44:1676-1681.

Joundi RA, Adekanye J, Leung AA, Ronksley P, Smith EE, Rebchuk AD, Field TS, Hill MD, Wilton SB, Bresee LC. Health State Utility Values in People With Stroke: A Systematic Review and Meta-Analysis. J Am Heart Assoc. 2022 Jul 5;11(13):e024296. doi: 10.1161/JAHA.121.024296. Epub 2022 Jun 22. PMID: 35730598; PMCID: PMC9333363.

Kahwati LC, Asher GN, Kadro ZO, Keen S, Ali R, Coker-Schwimmer E, Jonas DE. Screening for Atrial Fibrillation: Updated Evidence Report and Systematic Review for the US Preventive Services Task Force. JAMA. 2022 Jan 25;327(4):368-383. doi: 10.1001/jama.2021.21811. PMID: 35076660.

Kim AS, Nguyen-Huynh M, Johnston SC. A cost-utility analysis of mechanical thrombectomy as an adjunct to intravenous tissue-type plasminogen activator for acute large-vessel ischemic stroke. Stroke 2011;42:2013-2018.

Nguyen E, Egri F, Mearns ES, White CM, Coleman CI. Cost-effectiveness of high-dose edoxaban compared with adjusted-dose warfarin for stroke prevention in non-valvular atrial fibrillation patients. Pharmacotherapy 2016;36:488-495.

O’Brien CL, Gage BF. Costs and effectiveness of ximelagatran for stroke prophylaxis in chronic atrial fibrillation. JAMA 2005;293:699-706.

O’Brien JA, Patrick AR, Caro J. Estimates of direct medical costs for microvascular and macrovascular complications resulting from type 2 diabetes mellitus in the United States in 2000. Clin Ther 2003;25:1017-1038.

Patil CG, Long EF, Lansberg MG. Cost-effectiveness analysis of mechanical thrombectomy in acute ischemic stroke. J Neurosurg 2009;110:508-513.

Rebchuk AD, O’Neill ZR, Szefer EK, Hill MD, Field TS. Health Utility Weighting of the Modified Rankin Scale: A Systematic Review and Meta-analysis. *JAMA Netw Open.* 2020;3(4):e203767. doi:10.1001/jamanetworkopen.2020.3767

Rumberger JS, Hollenbeak CS, Kline D. Potential Costs and Benefits of Smoking Cessation: An Overview of the Approach to State Specific Analysis 2010 <https://www.lung.org/getmedia/aebd97aa-dc3b-4f62-8791-d6c891603d63/economic-benefits.pdf.pdf?ext=.pdf> (accessed: 2/17/2023)

Soeteman DI, Menzies NA, Pandya A. Would a large tPA trial for those 4.5 to 6.0 hours from stroke onset be good value for information? Value Health 2017;20:894-901

**Appendix C. Sensitivity Analyses**

| Table C1. Parameters in Sensitivity Analysis | | | | |
| --- | --- | --- | --- | --- |
|  | Base value | Lower Bound | Upper Bound |  |
| Parameters related to treatment effect |  |  |  |  |
| Log(hazard ratio) related to 1 unit of lipid ratio (Total cholesterol/HDL) | 0.113 | 0.064 | 0.162 | [1] |
| Log(hazard ratio) related to 1% of increase in A1c | 0.128 | 0.046 | 0.210 |  |
| Log(hazard ratio) related to 10 unit of increase in SBP | 0.276 | 0.194 | 0.358 |  |
| Relative risk for effect of Warfarin | 0.32 | 0.20 | 0.41 | [2] |
| Relative risk for effect of Aspirin | 0.88 | 0.79 | 0.97 | [3] |
| Cost (2014 US dollar) |  |  |  |  |
| Cost of Insulin treatment | Basal insulin: $5,504 per year  Intensive insulin: $16,488 per year | Basal insulin: $360 per year  Intensive insulin: $720 per year |  | [4] |

References

1. Clarke PM, Gray AM, Briggs A, Farmer AJ, Fenn P, Stevens RJ, Matthews DR, Stratton IM, Holman RR; UK Prospective Diabetes Study (UKDPS) Group. A model to estimate the lifetime health outcomes of patients with type 2 diabetes: the United Kingdom Prospective Diabetes Study (UKPDS) Outcomes Model (UKPDS no. 68). Diabetologia. 2004 Oct;47(10):1747-59. doi: 10.1007/s00125-004-1527-z. Epub 2004 Oct 27. PMID: 15517152.
2. Kahwati LC, Asher GN, Kadro ZO, Keen S, Ali R, Coker-Schwimmer E, Jonas DE. Screening for Atrial Fibrillation: Updated Evidence Report and Systematic Review for the US Preventive Services Task Force. JAMA. 2022 Jan 25;327(4):368-383. doi: 10.1001/jama.2021.21811. PMID: 35076660.
3. ASCEND Study Collaborative Group; Bowman L, Mafham M, Wallendszus K, Stevens W, Buck G, Barton J, Murphy K, Aung T, Haynes R, Cox J, Murawska A, Young A, Lay M, Chen F, Sammons E, Waters E, Adler A, Bodansky J, Farmer A, McPherson R, Neil A, Simpson D, Peto R, Baigent C, Collins R, Parish S, Armitage J. Effects of Aspirin for Primary Prevention in Persons with Diabetes Mellitus. N Engl J Med. 2018 Oct 18;379(16):1529-1539. doi: 10.1056/NEJMoa1804988. Epub 2018 Aug 26. PMID: 30146931.

Suran M. All 3 Major Insulin Manufacturers Are Cutting Their Prices-Here's What the News Means for Patients With Diabetes. JAMA. 2023 Apr 25;329(16):1337-1339. doi: 10.1001/jama.2022.11688. PMID: 36988974.

| Table C2. Sensitivity Analysis for Projected Average 10-year Outcomes of Primary Prevention Strategies for Patients with Type 2 Diabetes and No History of Stroke at Age 45 Years and Older (2018-2028). (All Enhanced vs. Status Quo) | | | | | | |
| --- | --- | --- | --- | --- | --- | --- |
|  | Change in individual stroke cost ($) | Change in QALY (Stroke related) | ICER  ($ per QALY gained) | Incremental Life-Years | Incremental stroke-related QALYs | Incremental NHB (in QALYs) |
| **Anti-coagulant treatment** |  |  |  |  |  |  |
| Base analysis (NOAC) | 807 | 0.0053 | 139,453 | 148,406 | 99,397 | -39,215 |
| Using warfarin instead of NOAC 1† | -42 | 0.0041 | Cost saving | 111,240 | 77,404 | 85,237 |
| Using warfarin instead of NOAC 2‡ | 267 | 0.0009 | 312,847 | 24,395 | 15,986 | -34,026 |
|  |  |  |  |  |  |  |
| **Aspirin treatment** |  |  |  |  |  |  |
| Base analysis | -324 | 0.0046 | Cost saving | 145,130 | 86,035 | 140,617 |
| Upper bound of efficacy | -516 | 0.0071 | Cost saving | 220,638 | 133,455 | 230,288 |
| Lower bound of efficacy | -45 | 0.0021 | Cost saving | 74,785 | 40,230 | 40,697 |
|  |  |  |  |  |  |  |
| **Well-controlled A1c** |  |  |  |  |  |  |
| Base analysis | 13,405 | -0.0021 | N/A | 330,059 | -38,479 | -2,551,859 |
| Upper bound of efficacy | 13,255 | -0.00062 | 77,872,999 | 395,692 | 3,191 | -2,481,840 |
| Lower bound of efficacy | 13,547 | -0.0039 | N/A | 291,714 | -64,462 | -2,604,370 |
| Lower cost of insulin treatment | 6,618 | -0.0019 | N/A | 338,099 | -35,342 | -1,276,077 |
|  |  |  |  |  |  |  |
| **Well-controlled BP** |  |  |  |  |  |  |
| Base analysis | -742 | 0.0175 | Cost saving | 616,506 | 327,324 | 466,508 |
| Upper bound of efficacy | -942 | 0.020 | Cost saving | 690,998 | 376.954 | 553,625 |
| Lower bound of efficacy | -585 | 0.0152 | Cost saving | 551,720 | 284,832 | 394,463 |
|  |  |  |  |  |  |  |
| **Statin treatment** |  |  |  |  |  |  |
| Base analysis | -51.9 | 0.0669 | Cost saving | 2,508,024 | 1,254,064 | 1,263,801 |
| Lower bound of efficacy | -103.9 | 0.0675 | Cost saving | 2,524,450 | 1,264,808 | 1,284,287 |
| Upper bound of efficacy | -10.4 | 0.0661 | Cost saving | 2,483,451 | 1,238,789 | 1,240,748 |
| † Assume risk reduction of warfarin treatment is 68% based on meta-analysis of clinical trials (Kahwati LC, Asher GN, Kadro ZO, Keen S, Ali R, Coker-Schwimmer E, Jonas DE. Screening for Atrial Fibrillation: Updated Evidence Report and Systematic Review for the US Preventive Services Task Force. JAMA. 2022 Jan 25;327(4):368-383. doi: 10.1001/jama.2021.21811. PMID: 35076660.)  ‡ Assume risk reduction of warfarin treatment is 22% based on observational studies. (Darkow T, Vanderplas AM, Lew KH, Kim J, Hauch O: Treatment patterns and real-world effectiveness of warfarin in nonvalvular atrial fibrillation within a managed care system. CMRO 2005, 21:1583–1594.) | | | | | | |

| Table C3. Projected 10-year Cost-effectiveness of Primary Prevention Strategies for Patients with Type 2 Diabetes and No History of Stroke at Age 45 Years and Older (2018-2028).* (50% Enhanced vs. Status Quo) | | | | | | |
| --- | --- | --- | --- | --- | --- | --- |
| Strategy | Stroke events averted No. (95 % UI) \|\|\|\| | Stroke attributed death averted No. (95 % UI) \|\|\|\| | Incremental total stroke cost  (95 % UI) \|\|\|\|  *Millions of $* | Incremental stroke-related QALYs (95 % UI) \|\|\|\| | Incremental NHB (in QALYs) (95 % UI) \|\|\|\| | ICER  ($/QALY) |
| Well controlled HbA1c§ | 15,812 | 6,603 | 135,526 | -25,700 | -1,380,957 | NA |
|  | (13,900 to 1,7723) | (5,335 to 7,871) | (134,836 to 136,216) | (-43,120 to -8,280) | (-1,399,694 to -1,362,221) |  |
| Well controlled BP‡ | 40,808 | 17,668 | -7,233 | 180,993 | 253,323 | Cost Saving |
|  | (38,902 to42,713) | (16,441 to 18,926) | (-7,931 to -6,535) | (163,560 to 198,425) | (234,546 to 272,099) |  |
| Statin treatment\|\| | 9,462 | 2,617 | -4,956 | 578,681 | 628,240 | Cost Saving |
|  | (7,547 to 11,377) | (1,350 to 3,883) | (-5,656 to -4,255) | (561,353 to 596,009) | (609,550 to 646,930) |  |
| Aspirin treatment¶ | 26,340 | 11,119 | -4,626 | 33,896 | 80,155 | Cost Saving |
|  | (24,435 to 28,245) | (9,858 to 12,380) | (-5,324 to -3,928) | (16,449 to 51,342) | (61,365 to 98,945) |  |
| Smoking cessation** | 4,282 | 1,111 | 132 | 10,740 | 9,417 | 12,313 |
|  | (2,367 to 6,198) | (158 to 2,380) | (-567 to 832) | (-6,778 to 28,258) | (-9,446 to 28,280) |  |
| Weight Loss† | 30,015 | 11,754 | 26,973 | 275,165 | 5,432 | 98,026 |
|  | (28,111 to 31,919) | (10,493 to 13,015) | (26,275 to 27,671) | (257,671 to 292,658) | (-13,404 to 24,267) |  |
| NOAC treatment‡‡ | 41,338 | -14,443 | 7,634 | 38,258 | -38,082 | 199,539 |
|  | (39,333 to 43,242) | (13,180 to 15,705) | (6,935 to 8,333) | (20,787 to 55,729) | (-56,899 to -19,265) |  |
| Multiple preventions 1^§§^ | 36,951 | 34,752 | -12,224 | 804,657 | 926,799 | Cost Saving |
|  | (35,668 to 38,234) | (32,257 to 34,752) | (-12,908 to -11,520) | (793,004 to 813,270) | (915,738 to 937,859) |  |
| * Data were calculated with the use of the Michigan Model for Diabetes on the basis of a simulation of the Recommendation of ADA (2022), AHA/ASA (2014) for preventing stroke in type 2 diabetes patients. A status quo simulation provided a projection of stroke events, costs, and quality-adjusted life-years (QALYs) for the US adult population between the ages of 45 years and older during the period from 2018 through 2028, on the assumptions that 1) the adherence level to each of the stroke prevention recommendation remain at the level observed in 2015-2018 (Table 1). ICER denotes incremental cost-effectiveness ratio.  † Weight Loss: 50% of patients with BMI > 25 at the beginning of simulation period attend weight loss programs and all lost 5% of weight  ‡ Well Controlled blood pressure (BP): 50% of patients who are not compliance to medication enhancement for blood pressure with enhancement threshold in the status-quo scenario become compliant.  § Well Controlled A1c: 50% of patients who are not compliance to medication enhancement with enhancement threshold in the status-quo scenario become compliant.  ¶ Aspirin treatment: All patients whose 10-year ASCVD risk >10% start taking aspirin in the year when this criteria is met  \|\| Statin treatment: 50%% patients who are eligible for taking statin but not taking statin in the study population start take statin at the beginning of simulation period.  ** Smoking Cessation: 50% of all smokers attend behavior intervention for smoking cessation and 12% successfully quit smoking at the beginning of simulation period.  †† Warfarin treatment: 50% of patients with atrial fibrillation (AFib) at high risk for stroke (defined as a CHA_2_DS_2_-VASc score >=2) start taking warfarin in the year when this criteria is met.  ‡‡ NOAC treatment: 50% patients with atrial fibrillation (AFib) at high risk for stroke (defined as a CHA_2_DS_2_-VASc score >=2) start taking NOAC in the year when this criteria is met.  §§ Implementing two cost-saving strategies (well controlled BP, aspirin treatment0) and two highly cost-effective strategies (smoking cessation and statin treatment).  ¶¶ Newly treated patients number for scenarios enhancing single prevention strategies only consider treatment related to the enhanced prevention; Newly treated patients number for scenarios enhancing multiple prevention strategies consider treatments related to all the enhanced preventions.  §§ Implementing two cost-saving strategies (well controlled BP, aspirin treatment, statin treatment) and one highly cost-effective strategy (smoking cessation).  \|\|\|\| 95% UI are from 100,000 simulations. | | | | | | |

| Table C4. Projected 10-year Cost-effectiveness of Primary Prevention Strategies for Patients with Type 2 Diabetes and No History of Stroke at Age 45 Years and Older (2018-2028).* (25% Enhanced vs. Status Quo) | | | | | | |
| --- | --- | --- | --- | --- | --- | --- |
| Strategy | Stroke events averted No. (95 % UI) \|\|\|\| | Stroke attributed death averted No. (95 % UI) \|\|\|\| | Incremental total stroke cost  (95 % UI) \|\|\|\|  *Millions of $* | Incremental stroke-related QALYs (95 % UI) \|\|\|\| | Incremental NHB (in QALYs) (95 % UI) \|\|\|\| | ICER  ($/QALY) |
| Well controlled HbA1c§ | 6,186 | 2,519 | 76,688 | -27,546 | -794,422 | NA |
|  | (4,273 to 8,100) | 1,253 to 3,786 | (75,991 to 77,384) | (-45,038 to -10,055) | (-813,250 to -775,595) |  |
| Well controlled BP‡ | 21,625 | 9,491 | -7,654 | 102,419 | 178,958 | Cost Saving |
|  | (19,713 to 23,537) | 8,226 to 10,756 | (-8,354 to -6,954) | (84,954 to 119,883) | (160,144 to 197,772) |  |
| Statin treatment\|\| | 3,698 | 696 | 3,020 | 220,503 | 190303 | 10,073 |
|  | (1,783 to 5,613) | -571 to 1,964 | (2,321 to 3,719) | (203,095 to 237,912) | (171545 to 209062) |  |
| Aspirin treatment¶ | 9,661 | 3,647 | -5,289 | 4,624 | 57,515 | Cost Saving |
|  | (7,749 to 11,573) | 2,381 to 4,912 | (-5,988 to -4,590) | (-12,887 to 22,135) | (38,660 to 76,369) |  |
| Smoking cessation** | 2,519 | 860 | 69 | 6,211 | 5,517 | 11,175 |
|  | (607 to 4,431) | -405 to 2,125 | (-629 to 768) | (-11,272 to 23,693) | (-13,311 to 24,344) |  |
| Weight Loss† | 14,157 | 5,593 | 13,659 | 136,014 | -571 | 100,420 |
|  | (12,247 to 16,067) | 4,330 to 6,856 | (12,959 to 14,359) | (118,495 to 153,534) | (-19,437 to 18,295) |  |
| NOAC treatment‡‡ | 18,741 | 6,541 | 2,514 | 11,354 | -13,782 | 221,383 |
|  | (16,825 to 20,656) | 5,274 to 7,809 | (1,812 to 3,215) | (-6,100 to 28,809) | (-32,593 to 5,029) |  |
| Multiple preventions 1^§§^ | 19,414 | 17,986 | -8,703 | 404,278 | 491,306 | Cost Saving |
|  | (18,125 to 20,703) | (16,729 to 19,244) | (-8,005 to -9,401) | (395,596 to 412,961) | (480,164 to 502,448) |  |
| * Data were calculated with the use of the Michigan Model for Diabetes on the basis of a simulation of the Recommendation of ADA (2022), AHA/ASA (2014) for preventing stroke in type 2 diabetes patients. A status quo simulation provided a projection of stroke events, costs, and quality-adjusted life-years (QALYs) for the US adult population between the ages of 45 years and older during the period from 2018 through 2028, on the assumptions that 1) the adherence level to each of the stroke prevention recommendation remain at the level observed in 2015-2018 (Table 1). ICER denotes incremental cost-effectiveness ratio.  † Weight Loss: 50% of patients with BMI > 25 at the beginning of simulation period attend weight loss programs and all lost 5% of weight  ‡ Well Controlled blood pressure (BP): 50% of patients who are not compliance to medication enhancement for blood pressure with enhancement threshold in the status-quo scenario become compliant.  § Well Controlled A1c: 50% of patients who are not compliance to medication enhancement with enhancement threshold in the status-quo scenario become compliant.  ¶ Aspirin treatment: All patients whose 10-year ASCVD risk >10% start taking aspirin in the year when this criteria is met  \|\| Statin treatment: 50%% patients who are eligible for taking statin but not taking statin in the study population start take statin at the beginning of simulation period.  ** Smoking Cessation: 50% of all smokers attend behavior intervention for smoking cessation and 12% successfully quit smoking at the beginning of simulation period.  †† Warfarin treatment: 50% of patients with atrial fibrillation (AFib) at high risk for stroke (defined as a CHA_2_DS_2_-VASc score >=2) start taking warfarin in the year when this criteria is met.  ‡‡ NOAC treatment: 50% patients with atrial fibrillation (AFib) at high risk for stroke (defined as a CHA_2_DS_2_-VASc score >=2) start taking NOAC in the year when this criteria is met.  §§ Implementing two cost-saving strategies (well controlled BP, aspirin treatment0) and two highly cost-effective strategies (smoking cessation and statin treatment).  ¶¶ Newly treated patients number for scenarios enhancing single prevention strategies only consider treatment related to the enhanced prevention; Newly treated patients number for scenarios enhancing multiple prevention strategies consider treatments related to all the enhanced preventions.  §§ Implementing well controlled BP, aspirin treatment, statin treatment, and smoking cessation.  \|\|\|\| 95% UI are from 100,000 simulations. | | | | | | |

| Table C5. Projected life-time Cost-effectiveness of Primary Prevention Strategies for Patients with Type 2 Diabetes and No History of Stroke at Age 45 Years and Older (2018-2068).* (Optimal vs. Status Quo) | | | | | | |
| --- | --- | --- | --- | --- | --- | --- |
| Strategy | Stroke events averted No. (95 % UI) \|\|\|\| | Stroke attributed death averted No. (95 % UI) \|\|\|\| | Incremental total stroke cost  (95 % UI) \|\|\|\|  *Millions of $* | Incremental stroke-related QALYs (95 % UI) \|\|\|\| | Incremental NHB (in QALYs) (95 % UI) \|\|\|\| | ICER  ($/QALY) |
| Well controlled HbA1c§ | 97,716 | 41,011 | 438,202 | 928,143 | -3,453,876 | 472,127 |
|  | (94,762 to 100,670) | (38,779 to 43,242) | (436,259 to 440,145) | (874,862 to 981,424) | (-3,510,589 to -3,397,163) |  |
| Well controlled BP‡ | 45,722 | 24,658 | -2,587 | 974,985 | 1,000,858 | Cost saving |
|  | (42,753 to 48,690) | (22,420 to 26,896) | (-4,540 to -634) | (921,689 to 1,028,280) | (944,097 to 1,057,619) |  |
| Statin treatment\|\| | -95,417 | -65,772 | 174,539 | 6,324,616 | 4,579,228 | 27,597 |
|  | (-98,404 to -92,429) | (-68,035 to -63,510) | (172,576 to 176,502) | (6,271,696 to 6,377,536) | (4,522,784 to 4,635,671) |  |
| Aspirin treatment¶ | 181,366 | 105,078 | -11,574 | 720,837 | 836,579 | Cost saving |
|  | (178,424 to 184,309) | (102,864 to 107,293) | (-13,517 to -9,631) | (667,444 to 774,231) | (779,759 to 893,398) |  |
| Smoking cessation** | 21,462 | 9,775 | 5,477 | 237,726 | 182,960 | 23,037 |
|  | (18,491 to 24,433) | (7,530 to 12,020) | (3,521 to 7,432) | (184,388 to 291,063) | (126,152 to 239,768) |  |
| Weight Loss† | 160,040 | 70,349 | 152,153 | 2,765,269 | 1,243,743 | 55,023 |
|  | (157,084 to 162,996) | (68,122 to 72,577) | (150,187 to 154,118) | (2,711,757 to 2,818,780) | (1,186,737 to 1,300,750) |  |
| NOAC treatment‡‡ | 101,481 | 45,031 | 11,875 | 398,096 | 279,350 | 29,828 |
|  | (98,519 to 104,444) | (42,797 to 47,265) | (9,917 to 13,832) | (344,789 to 451,404) | (222,563 to 336,138) |  |
| * Data were calculated with the use of the Michigan Model for Diabetes on the basis of a simulation of the Recommendation of ADA (2022), AHA/ASA (2014) for preventing stroke in type 2 diabetes patients. A status quo simulation provided a projection of stroke events, costs, and quality-adjusted life-years (QALYs) for the US adult population between the ages of 45 years and older during the period from 2018 through 2028, on the assumptions that 1) the adherence level to each of the stroke prevention recommendation remain at the level observed in 2015-2018 (Table 1). ICER denotes incremental cost-effectiveness ratio.  † Weight Loss: 50% of patients with BMI > 25 at the beginning of simulation period attend weight loss programs and all lost 5% of weight  ‡ Well Controlled blood pressure (BP): 50% of patients who are not compliance to medication enhancement for blood pressure with enhancement threshold in the status-quo scenario become compliant.  § Well Controlled A1c: 50% of patients who are not compliance to medication enhancement with enhancement threshold in the status-quo scenario become compliant.  ¶ Aspirin treatment: All patients whose 10-year ASCVD risk >10% start taking aspirin in the year when this criteria is met  \|\| Statin treatment: 50%% patients who are eligible for taking statin but not taking statin in the study population start take statin at the beginning of simulation period.  ** Smoking Cessation: 50% of all smokers attend behavior intervention for smoking cessation and 12% successfully quit smoking at the beginning of simulation period.  †† Warfarin treatment: 50% of patients with atrial fibrillation (AFib) at high risk for stroke (defined as a CHA_2_DS_2_-VASc score >=2) start taking warfarin in the year when this criteria is met.  ‡‡ NOAC treatment: 50% patients with atrial fibrillation (AFib) at high risk for stroke (defined as a CHA_2_DS_2_-VASc score >=2) start taking NOAC in the year when this criteria is met.  §§ Implementing two cost-saving strategies (well controlled BP, aspirin treatment0) and two highly cost-effective strategies (smoking cessation and statin treatment).  ¶¶ Newly treated patients number for scenarios enhancing single prevention strategies only consider treatment related to the enhanced prevention; Newly treated patients number for scenarios enhancing multiple prevention strategies consider treatments related to all the enhanced preventions.  \|\|\|\| 95% UI are from 100,000 simulations. | | | | | | |

**Appendix D. Impact inventory for components considered in the cost-effectiveness analyses**

| Type of impact | Included in the reference case analysis from each perspective | | Notes on sources of evidence |
| --- | --- | --- | --- |
|  | Health care sector | Societal |  |
| Formal health care sector |  |  |  |
| Health |  |  |  |
| Health outcomes (effects) |  |  |  |
| Longevity effects | Yes | NA | MMD |
| Health-related quality-of-life effects | Yes | NA | MMD (utilities from published literature) |
| Other health effects | Yes | NA | MMD |
| Medical costs |  |  |  |
| Paid for by third-party payers | Yes | NA | MMD (costs from published literature) |
| Paid for by patients out-of-pocket | No | NA |  |
| Future related medical costs | Yes | NA | MMD (costs from published literature) |
| Future unrelated medical costs | No | NA |  |
| Informal health care sector |  |  |  |
| Health |  |  |  |
| Patient-time costs | NA | NA |  |
| Unpaid caregiver-time costs | NA | NA |  |
| Transportation costs | NA | NA |  |
| Non–health care sectors |  |  |  |
| Productivity | NA | NA |  |
| Consumption | NA | NA |  |
| Social services | NA | NA |  |
| Legal or criminal justice | NA | NA |  |
| Education | NA | NA |  |
| Housing | NA | NA |  |
| Environment | NA | NA |  |

Abbreviations: NA, not applicable; MMD, Michigan Model for Diabetes.

# Appendix E. CHEERS 2022 Checklist

| **Topic** | **No.** | **Item** | **Location where item is reported** |
| --- | --- | --- | --- |
| **Title** |  |  |  |
|  | 1 | Identify the study as an economic evaluation and specify the interventions being compared. | title page |
| **Abstract** |  |  |  |
|  | 2 | Provide a structured summary that highlights context, key methods, results, and alternative analyses. | abstract page |
| **Introduction** |  |  |  |
| **Background and objectives** | 3 | Give the context for the study, the study question, and its practical relevance for decision making in policy or practice. | Introduction Section |
| **Methods** |  |  |  |
| **Health economic analysis plan** | 4 | Indicate whether a health economic analysis plan was developed and where available. | NA |
| **Study population** | 5 | Describe characteristics of the study population (such as age range, demographics, socioeconomic, or clinical characteristics). | Base-case analysis paragraph in the Method Section and Appendix D |
| **Setting and location** | 6 | Provide relevant contextual information that may influence findings. | Introduction Section |
| **Comparators** | 7 | Describe the interventions or strategies being compared and why chosen. | Introduction Section and Base-case analysis paragraph in the Method Section |
| **Perspective** | 8 | State the perspective(s) adopted by the study and why chosen. | Base-case analysis paragraph in the Method Section |
| **Time horizon** | 9 | State the time horizon for the study and why appropriate. | Health outcomes and Costs paragraphs in the Methods Section |
| **Discount rate** | 10 | Report the discount rate(s) and reason chosen. | Base-case analysis paragraph in the Method Section |
| **Selection of outcomes** | 11 | Describe what outcomes were used as the measure(s) of benefit(s) and harm(s). | Health outcomes and Costs paragraphs in the Methods Section |
| **Measurement of outcomes** | 12 | Describe how outcomes used to capture benefit(s) and harm(s) were measured. | Health outcomes and Costs paragraphs in the Methods Section |
| **Valuation of outcomes** | 13 | Describe the population and methods used to measure and value outcomes. | Base-case analysis paragraphs, health outcomes, and costs paragraphs in the Method Section |
| **Measurement and valuation of resources and costs** | 14 | Describe how costs were valued. | Cost paragraph in the Method Section and in the Supplementary materials |
| **Currency, price date, and conversion** | 15 | Report the dates of the estimated resource quantities and unit costs, plus the currency and year of conversion. | Base-case analysis paragraph in the Method Section |
| **Rationale and description of model** | 16 | If modelling is used, describe in detail and why used. Report if the model is publicly available and where it can be accessed. | The Michigan Model for Diabetes paragraphs in the Methods Section |
| **Analytics and assumptions** | 17 | Describe any methods for analysing or statistically transforming data, any extrapolation methods, and approaches for validating any model used. | The Michigan Model for Diabetes paragraphs in the Methods Section |
| **Characterising heterogeneity** | 18 | Describe any methods used for estimating how the results of the study vary for subgroups. | NA |
| **Characterising distributional effects** | 19 | Describe how impacts are distributed across different individuals or adjustments made to reflect priority populations. | NA |
| **Characterising uncertainty** | 20 | Describe methods to characterise any sources of uncertainty in the analysis. | Sensitivity Analyses in the Methods Section |
| **Approach to engagement with patients and others affected by the study** | 21 | Describe any approaches to engage patients or service recipients, the general public, communities, or stakeholders (such as clinicians or payers) in the design of the study. | NA |
| **Results** |  |  |  |
| **Study parameters** | 22 | Report all analytic inputs (such as values, ranges, references) including uncertainty or distributional assumptions. | The Michigan Model for Diabetes paragraphs in the Methods Section and Supplementary materials |
| **Summary of main results** | 23 | Report the mean values for the main categories of costs and outcomes of interest and summarise them in the most appropriate overall measure. | Results section |
| **Effect of uncertainty** | 24 | Describe how uncertainty about analytic judgments, inputs, or projections affect findings. Report the effect of choice of discount rate and time horizon, if applicable. | Sensitivity Analyses paragraph in the Results Section, last paragraph in the Discussion Section. |
| **Effect of engagement with patients and others affected by the study** | 25 | Report on any difference patient/service recipient, general public, community, or stakeholder involvement made to the approach or findings of the study | NA |
| **Discussion** |  |  |  |
| **Study findings, limitations, generalisability, and current knowledge** | 26 | Report key findings, limitations, ethical or equity considerations not captured, and how these could affect patients, policy, or practice. | Discussion section |
| **Other relevant information** |  |  |  |
| **Source of funding** | 27 | Describe how the study was funded and any role of the funder in the identification, design, conduct, and reporting of the analysis | Funding/Support |
| **Conflicts of interest** | 28 | Report authors conflicts of interest according to journal or International Committee of Medical Journal Editors requirements. | Conflict of interest disclosure |

*From:* Husereau D, Drummond M, Augustovski F, et al. Consolidated Health Economic Evaluation Reporting Standards 2022 (CHEERS 2022) Explanation and Elaboration: A Report of the ISPOR CHEERS II Good Practices Task Force. Value Health 2022;25. <doi:10.1016/j.jval.2021.10.008>

**Appendix F. Baseline Characteristics of the Simulation Population**

| Table F1: Baseline characteristics in T2DM patients ≥45 years of age without stroke history (NHANES 2015-2018) N=1232 | | |
| --- | --- | --- |
|  | Unweighted  Mean (SD) or % | Weighted  Mean (SE) or % |
| Demographics |  |  |
| Age | 65.1 (9.8) | 64.0 (0.41) |
| Male | 55.8 | 56.1 |
| Race |  |  |
| *Mexican American* | 19.5 | 9.3 |
| *Non-Hispanic Asian* | 12.6 | 6.7 |
| *Non-Hispanic Black* | 22.7 | 12.0 |
| *Non-Hispanic White* | 29.7 | 61.2 |
| *Other Hispanic* | 11.8 | 5.8 |
| *Other race* | 3.8 | 4.8 |
| DM duration | 11.9 (9.3) | 11.0 (0.37) |
| Risk Factors |  |  |
| Smoking | 18.6 | 18.9 |
| A1c (%) | 7.44 (1.63) | 7.29 (0.06) |
| BMI (kg/m^2^) | 32.1 (7.4) | 32.7 (0.32) |
| BMI > 25 kg/m^2^ | 85.9 | 89.2 |
| SBP (mmHg) | 133.4 (19.7) | 130.6 (0.80) |
| DBP (mmHg) | 67.7 (14.2) | 68.6 (0.57) |
| HDL (mmol/L) | 1.26 (0.38) | 1.23 (0.02) |
| LDL (mmol/L) | 2.63 (0.97) | 2.64 (0.05) |
| TG (mmol/L) | 1.94 (1.60) | 2.10 (0.08) |
| Total Cholesterol | 4.56 (1.13) | 4.57 (0.05) |
| Medication use |  |  |
| Aspirin | 2.4 | 1.8 |
| Clopidogrel | 5.8 | 6.1 |
| ACEI/ARB | 64.9 | 66.2 |
| Beta blocker | 31.3 | 30.2 |
| Statin | 58.5 | 60.0 |
| Warfarin | 2.8 | 3.4 |
| Non-Vitamin K antagonist oral anticoagulants (NOAC) | 2.1 | 1.7 |
| Anti-glycaemia medications |  |  |
| *0 (No OAD and no insulin)* | 16.8 | 15.2 |
| *1 (1 OAD and no insulin)* | 40.4 | 44.0 |
| *>1 (>1 OAD and no insulin)* | 23.9 | 22.8 |
| *Basal* | 10.6 | 10.7 |
| *Intensive* | 8.3 | 7.3 |
| Number of antihypertensive drugs |  |  |
| *1* | 44.1 | 46.1 |
| *2* | 22.3 | 22.0 |
| *3* | 7.0 | 5.5 |
| *4* | 1.5 | 1.5 |
| *5* | 0.02 | 0.08 |
| Medical history |  |  |
| Angina | 3.3 | 4.3 |
| Myocardial infarction (w/o HF) | 6.1 | 7.5 |
| Heart failure (w/o MI) | 5.1 | 4.6 |
| MI and HF | 4.7 | 4.0 |
| Dialysis | 1.2 | 0.65 |

**Appendix G. Additional Base-case Analysis Results**

Figure G1. Achievement of well-controlled HbA1c, BMI, and BP, in the status-quo scenario and enhanced prevention strategies.
